# Liver transplantation in a boy with *TFAM* mutation associated mtDNA depletion syndrome

**DOI:** 10.1186/s13023-024-03487-1

**Published:** 2024-12-23

**Authors:** Jing Zhao, Lian Chen, Ni Wang, Xin-bao Xie

**Affiliations:** 1 The Center for Pediatric Liver Diseases, Children’s Hospital of Fudan University, 399 Wanyuan Road, Minhang District, Shanghai, 201102 China; 2 Department of Pathology, Children’s Hospital of Fudan University, Shanghai, China; 3 The Third Affiliated Hospital of Zunyi Medical University, 98 Fenghuang North Road, Huichan District, Zunyi, Guizhou China

**Keywords:** MtDNA depletion syndrome, Liver transplantation, TFAM, Neonatal liver failure

## Abstract

Mitochondrial transcription factor A (TFAM) deficiency may cause mtDNA depletion syndrome, which manifests as neonatal liver failure or primary ovarian insufficiency, hearing loss, seizures, and intellectual disability. Treatment focusing on symptomatic management, and the clinical prognosis remains poor. Here, we describe a novel case of *TFAM* mutation presenting with progressive neonatal cholestasis, hypoglycemia and abnormal amino acid profiling. The patient progressed to liver failure at 6 months of age but did not exhibit neurological involvement. No morphologic abnormalities were observed in muscle biopsy, while mtDNA copy number was reduced in comparison to age- and tissue-matched controls. After liver transplantation, liver biochemistries and blood amino acid profiling normalized three weeks later. Moreover, the boy was doing well post-transplant without any clinical concerns, and his development and neurological examination remain normal 33 months after liver transplantation. Our report suggests that liver transplantation appears to have a favorable profile in such patients.

**Supplementary Information:**

The online version contains supplementary material available at 10.1186/s13023-024-03487-1.

## Introduction

Human mitochondrial transcription factor A (TFAM) plays a central role in the organization, expression and maintenance of the mitochondrial genome [1, 2]. TFAM deficiency is associated with autosomal recessive mtDNA depletion syndromes. In 2016, two siblings were first reported to have mtDNA depletion syndrome caused by a homozygous missense variant (NM_003201.2, c.533C > T, p.Pro178Leu) in *TFAM*. They presented with intrauterine growth restriction, elevated transaminases, conjugated hyperbilirubinemia and hypoglycemia, which progressed to liver failure and death in early infancy [3]. Additional study showed that the affected individuals displayed variable phenotypes ranging from sensorineural hearing loss to primary ovarian insufficiency, accompanied by seizures and intellectual disability [4].

Although liver transplantation remains the only treatment option for liver failure in hepatocerebral mtDNA depletion syndromes; owing to the multi-organ involvement, liver transplantation in mitochondrial hepatopathy is controversial [5–8]. However, children with isolated hepatic disease have excellent 10 year survival rates with liver transplantation and, thus, it represents a potential therapeutic option [5]. Here, we describe a Chinese boy presenting with progressive neonatal cholestasis, and received liver transplantation later.

## Case description

A male Chinese infant, weighing 2.4 kg at birth, was delivered by cesarean section at 41 weeks of gestation with intrauterine growth restriction since 37 weeks. The parents were not consanguineous. Three days after birth, he developed persistent jaundice by receiving treatment with oral ursodeoxycholic acid, but his condition worsened. Panel sequencing showed a homozygous variant of c.553C > T (p.Pro178Leu) in *TFAM*. At the age of 4 months, he was admitted to our hospital for progressive jaundice.

His height (61 cm) was within normal limits (normal range: 60.5–69.1 cm), but weight (5.6 kg) was below normal range (normal range: 6.1–9.2 kg). Jaundice and splenomegaly were observed. Laboratory tests showed direct hyperbilirubinemia (total bilirubin level of 165 μmol/L, direct bilirubin level of 106.9 μmol/L), elevated aminotransferases, normal gama-glutamyl transpeptidase and coagulopathy (Table 1). Further work-up revealed hypoketo and hypoglycemia, with a slightly elevated lactic acid level peaking at 2.6 mmol/L (normal range: 0.5–1.6 mmol/L), abnormal amino acid profiling and mild hearing loss in the left ear. Ultrasonography showed poor liver texture with several space-occupying lesions.

**Table 1:** The laboratory investigations before and after living donor liver transplantation

	Pre-liver transplantation				Post-liver transplantation					
weeks	9	8	4	1	1	3	24	48	110	142
TB (5.1–17.1 μmol/L)	165	206.6	361.0	639.9	85.4	11.0	3.2	4.2	9.4	15
DB (0–6 μmol/L)	106.9	153.5	158.5	397.7	60.2	6.9	1.5	1.7	3.3	2.9
ALT (0–40 IU/L)	115.5	244.5	244.8	310.9	197.6	52.8	23.1	24.3	22.6	34
AST (0–40 IU/L)	401.5	982.2	691.0	515.6	120.0	46.9	33.1	42.1	36.7	46
ALP (IU/L)	1300	1080.5	694.5	916.5	192.0	147.3	322.3	383.3	373.7	377
GGT (7–50 IU/L)	101.2	71.6	45.1	30.7	36.4	43.8	10.8	10.8	18.3	29
TBA (0–10 μmol/L)	359.4	372.4	361.0	290.1	NA	3.9	8.4	8.4	20.8	12.3
Alb (35–55 g/L)	35.2	33.6	37.7	37.9	41.1	38.3	39.8	36.5	44.1	45.2
PT (11–14.5 s)	15	16.9	14.7	36	25.9	18	NA	NA	NA	NA
Lac (0.5–1.6 mmol/L)	2.6	2.3	1.8	3	1.3	NA	NA	NA	NA	NA
CK (0–164 IU/L)	122	NA	165	828	37	33	48	119	78	77
CK-MB (< 25 IU/L)	39.9	NA	30.8	38.4	12	18.8	43.2	54.4	40.0	27
Cr(μmol/L)	19.4	16.8	19.4	16.8	19.3	19.6	28.1	25.2	30.3	33
cTnl (1.5–19 ng/L)	NA	NA	NA	70.8	59.1	NA	NA	NA	NA	NA

*TB*, Total bilirubin; *DB*, Direct bilirubin; *ALT*, Alanine transaminase; *AST*, Aspartate transaminase; *ALP*, Alkaline phosphatase; *GGT*, Gamma glutamyl transpeptidase; *TBA*, Total bile acids; *Alb*, Albumin; *PT*, Prothrombin time; *Lac*, Lactic acid; *CK*, Creatine kinase; *CK-MB*, Creatine kinase isoenzyme; *cTnl*, Troponin

Neurological evaluation, including developmental and cognitive assessment, brain MRI, and electroencephalogram, displayed no obvious abnormality except for a thin corpus callosum with T2-hyperintense lesions of the knee (Supplementary figure S1). Cardiac ultrasound revealed mild bicuspid regurgitation. After the informed parental consent was obtained, we performed whole exome sequencing on the patient and his parents, and identified biallelic missense variants in the TFAM gene.

**Fig. 1:**
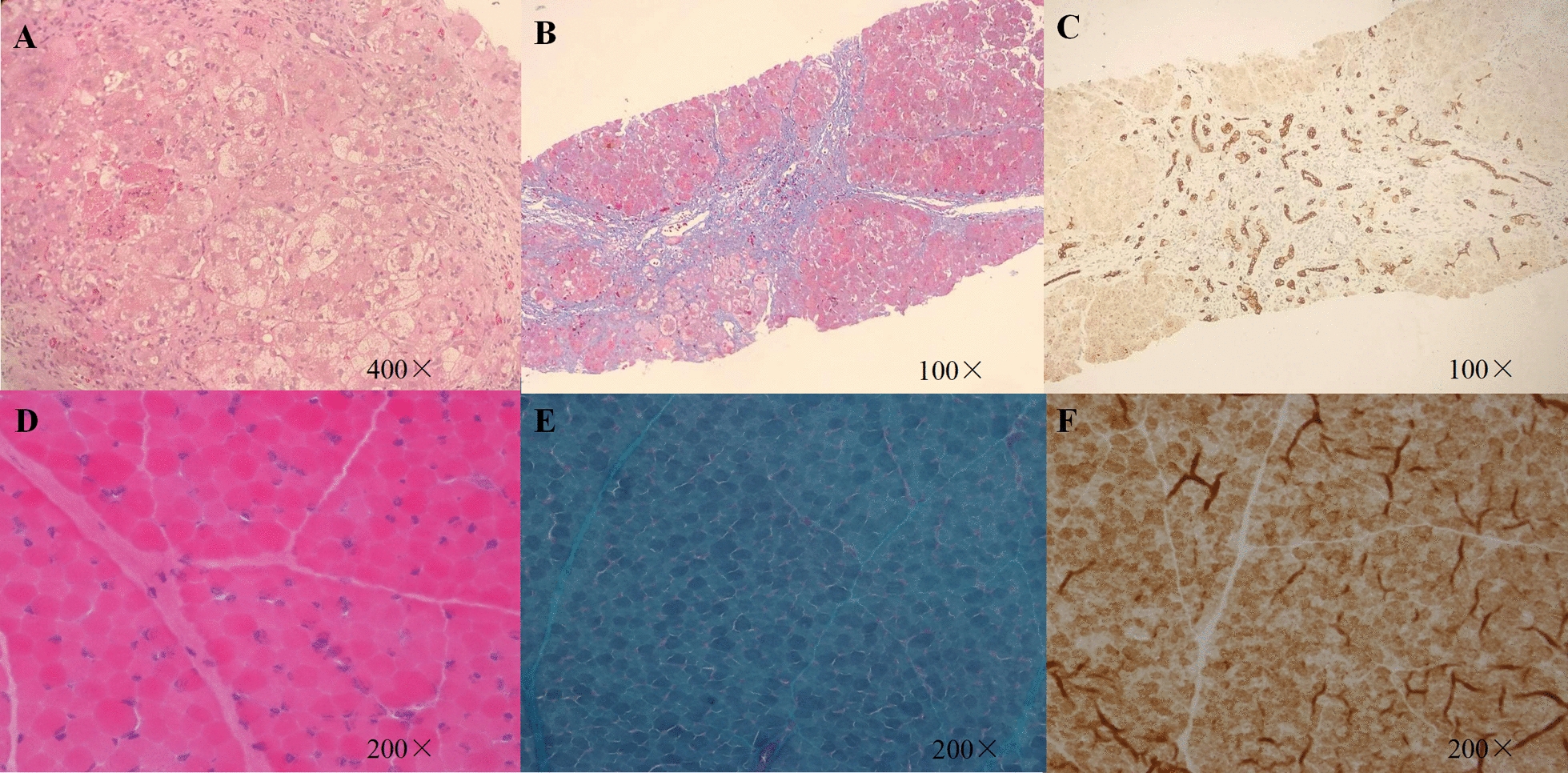
Liver biopsy and muscle biopsy. **A** intralobular cholestasis (hepatocellular and canalicular), giant-cell transformation of hepatocytes (HE). **B** Bridging fibrosis, pseudolobelia formation (Masson). **C**, Biliary ducts proliferation with irregularly arranged and vacuolar epithelial cells (CK 19). **D**, HE staining, the muscle fibers were of uniform size, with no typical degeneration, necrosis, or regeneration of muscle fibers, no aggregation of special blue particles, and no fragmented red fibers (RRF). **E**, Gomori Trichrome staining, no RRF or rod-shaped bodies were observed. **F**, The staining of succinate dehydrogenase (SDH) and cytochrome C oxidase (COX), there was no abnormal activity of the enzyme and no SSV phenomenon

The liver biopsy demonstrated intralobular cholestasis (hepatocellular and canalicular), giant-cell transformation of hepatocytes, bridging fibrosis, pseudolobelia formation, biliary ducts proliferation with irregularly arranged and vacuolar epithelial cells (Fig. 1). No morphologic abnormalities were noted in the muscle tissue (Fig. 1). Since the liver biopsy tissue was too small to test for mtDNA content, a test for mtDNA content was performed using a muscle sample by real-time quantitative PCR analysis [9]. The mtDNA copy number in the muscle (622 ± 102) was reduced compared to age and tissue matched controls (1746 ± 361).

This patient was treated with ursodeoxycholic acid (20 mg/kg/day, divided into two daily doses), fat-soluble vitamins, vitamin B1, B2, L-carnitine and coenzyme Q10. Due to recurrent hypoglycemia, he received continuous feeding with formulas enriched in medium chain triglycerides via a nasogastric tube. However, the patient’s jaundice and coagulopathy worsened overtime, along with anemia, thrombocytopenia and abnormal myocardial enzymes. No obvious abnormality was found in either the MRI of the brain or the cardiac ultrasound (Supplementary figure S1). Fortunately, he had normal development without muscle hypotonia or neurological symptoms.

The patient was listed for liver transplantation due to acute-on-chronic liver failure with a natural Pediatric End-Stage Liver Disease (PELD) score of 21 meaning that our patient would derive survival benefit early after liver transplantation, and underwent living donor liver transplantation at 6 months of age. He received induction immunosuppression with tacrolimus and methylprednisolone. His post-operative course was complicated with fungal septicemia, pneumonia, hypothyroidism, pancytopenia, pericardial effusion, abnormal cardiac ultrasound (left atrial and ventricular enlargement, mild to moderate mitral regurgitation) and increased myocardial enzymes: creatine kinase 3077U/L, creatine kinase isoenzyme 51.4U/L, cardiac tropnin I 59.1 μg/L). Myocardial enzymes normalized five days later, and liver biochemistries and cardiac ultrasound normalized three weeks later. He was discharged on postoperative day 25 on tecrolimus, corticosteroid and sodium levothyroxine.

Three months after liver transplantation, he received balloon dilatation for stenosis of the portal vein anastomosis and then warfarin treatment for 6 months. Cardiac ultrasound revealed slight thickening of the ventricular septum and the posterior wall of the left ventricle 10 months after liver transplantation. The boy is now 3 year and 3 months old and is doing well post-transplant with no clinical concerns. His development and neurological examination remain normal. His treatment consists only of oral tacrolimus (0.0125 mg/kg/day), and the results of routine liver function tests are normal. His growth is satisfactory (height = 99 cm, weight = 16.5 kg). Creatine kinase is normal with creatine kinase isoenzyme being slightly higher and cardiac ultrasound reveals mild tricuspid regurgitation (Table 1). No obvious abnormality is found in the head MRI scan and electroencephalogram in the waking state.

## Discussion

In this report, we described a patient with a homozygous mutation in *TFAM* who was referred to our hospital due to progressive neonatal cholestasis, hypoglycemia, abnormal amino acid profiling and mild hearing loss in the left ear. The boy progressed to liver failure requiring liver transplantation at the age of 6 months. After liver transplantation, he developed normally without any detectable neurological disorders.

Individuals with TFAM mutations exhibited two distinct groups of clinical manifestations: one characterized by intrauterine growth restriction, elevated transaminases, and cholestasis with progression to liver failure and death in early infancy; the other presenting with primary ovarian insufficiency, seizures, intellectual disability and hearing loss, apparently reflecting the difference of the two genotypes, p.Pro178Leu and p.Arg232Cys [3, 4]. Molecular modeling suggests that the p.Pro178Leu mutation influences promoter sequence recognition and the interaction between TFAM and the tether helix of POLRMT, thereby elucidating transcription initiation deficiency [10].

Although the shared biallelic missense pathogenic variant (p.Pro178Leu) between a patient reported in 2016 and our patient, the clinical phenotype observed in our patient lacked manifestations of myopathy or neuropathy.

Liver histology showed intralobular cholestasis, bridging fibrosis, biliary ducts proliferation and mildly microvascular steatosis. Although mtDNA copy number analysis in liver tissue was not performed, muscle biopsies showed a reduced mtDNA content (35.7%), which was consistent with the diagnosis of mtDNA depletion. Our patient exhibited normal development without muscle hypotonia or neurological symptoms and thus was considered to have isolated severe liver disease. He experienced fungal sepsis, pneumonia and intestinal infection after liver transplantation, which might be related to the regulation of innate immunity by mitochondria and the use of immunosuppressants.

During a 33 months follow-up period, no neurological manifestations was observed. Mild myocardial thickening occurred 10 months after liver transplantation and relieved later, but further follow-up was required. The decision to perform liver transplantation for individuals afflicted with this disease remains difficult, as neurological manifestations or myopathy may occur or worsen after liver transplantation, despite their absence before transplantation. Our study provides novel insight into the therapeutic approach for TFAM deficiency and suggests that liver transplantation appears a potentially favorable safety profile. Subsequent studies will be necessary to document the long-term implications of this approach on disease outcome.

## Supplementary Information

Supplementary material 1.

## Data Availability

All data generated or analyzed during this study are included in this published article and its supplementary information files.
