# Supplementary figures and images for: Liver transplantation in a boy with TFAM mutation associated mtDNA depletion syndrome
Source: Orphanet J Rare Dis. 2024 Dec 23;19:486. doi: 10.1186/s13023-024-03487-1 (PMC11668023; doi:10.1186/s13023-024-03487-1)

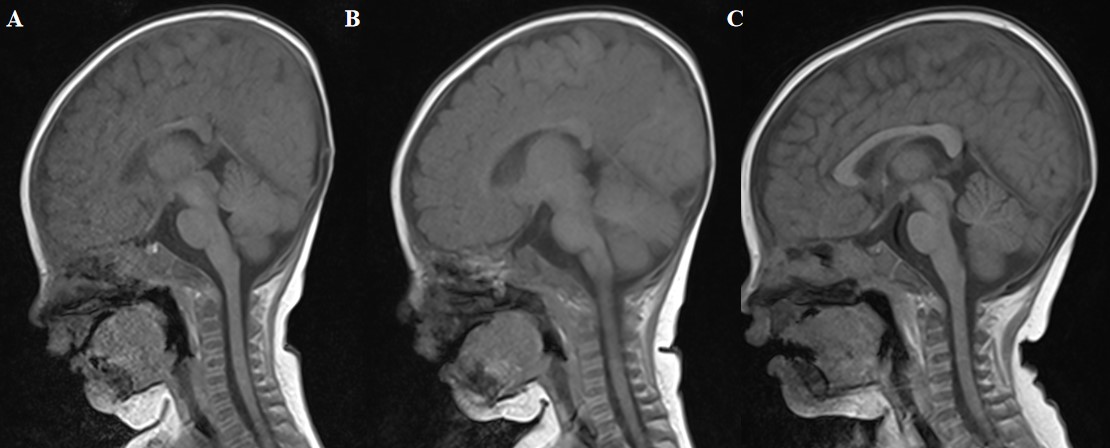

Supplement: Supplementary file 1 — Supplementary material 1. [file 13023_2024_3487_MOESM1_ESM.jpg]
